# Women’s communication self-efficacy and expectations of primary male partners’ cooperation in sexually transmitted infection treatment in Ho Chi Minh City, Vietnam: a cross-sectional study

**DOI:** 10.1186/s12889-016-2690-0

**Published:** 2016-01-13

**Authors:** Ly Thi-Hai Tran, Thanh Cong Bui, Christine M. Markham, Michael D. Swartz, Quan Minh Tran, Alan G. Nyitray, Thuy Thi-Thu Huynh, Lu-Yu Hwang

**Affiliations:** 1 Department of Epidemiology, School of Public Health, The University of Texas Health Science Center at Houston, Houston, TX USA; 2 Department of Behavioral Science, The University of Texas MD Anderson Cancer Center, Houston, TX USA; 3 Department of Health Promotion and Behavioral Sciences, School of Public Health, The University of Texas Health Science Center at Houston, Houston, TX USA; 4 Department of Biostatistics, School of Public Health, The University of Texas Health Science Center at Houston, Houston, TX USA; 5 Pham Ngoc Thach University of Medicine, Ho Chi Minh City, Vietnam; 6 Tu Du Hospital, Ho Chi Minh City, Vietnam

**Keywords:** STIs, Self-efficacy, Women, Partner, Vietnam

## Abstract

**Background:**

Effective control of sexually transmitted infections (STIs) depends on affected patients notifying their sexual partners, and partners following through with screening and treatment. Our study assessed high-risk-STI women’s confidence in STI-diagnosis-related communications with their primary male partners in Ho Chi Minh City, Vietnam, and determined associated characteristics of the women and their partners.

**Methods:**

We employed convenience and snowball sampling in a clinic-based setting to recruit 126 women from August to October 2013. All data were obtained from women’s self-report.

**Results:**

The proportions of participants who were “slightly confident” or “very confident” that they could disclose their STI positivity to partners, ask partners to have an STI examination or treatment, and give partners bacterial-STI medications were 70.3 %, 62.1 %, and 69.0 %, respectively. The proportions who perceived that their partners would be “very likely” to have an STI examination and to take STI medications were 16.2 % and 38.8 %, respectively. Significantly lower self-efficacy was observed in women who had a lower education level, who had ever traded sex, or whose primary partners were not husbands or fiancés.

**Conclusions:**

Our results suggest potential for piloting STI-partner-targeted interventions. To be effective, these programs should improve women’s self-efficacy and primary partners’ cooperation with screening and treatment.

## Background

In developing countries such as Vietnam, sexually transmitted infections (STIs) and their complications are one of the top five reasons that adults seek health care [1]. A study in five border provinces in Vietnam found that the infection rate of gonorrhea or chlamydia among female sex workers (FSWs) was 19.9 %, ranging from 11.3 % to 32.7 % [2]. Although a women’s risk of having an STI has been found to be associated with their primary male partner’s behavioral risk factors [3], still no partner-targeted STI prevention service is being developed in Vietnam. The current main strategy for controlling STIs is patient-based syndromic management, i.e., offering treatment for a single individual primarily based on clinical syndromes [4]. However, this strategy appears insufficient given that several studies have shown a high prevalence of recurrent STIs [5, 6]. The World Health Organization (2001) has recommended that STI care include notification of sexual partners and, in the case of a bacterial STI, partner treatment. Through additionally reaching asymptomatic sexual partners, partner-targeted interventions effectively prevent STI spread. Although partner notification can be done by health care professionals, a recent systematic review showed that the relative effect of provider-led partner referral was not consistent and was not significantly superior to other patient-led methods [7]. Therefore, in the context of Vietnam where healthcare providers are generally overloaded with clinical work, exploring the potential of other patient-led STI control methods is necessary. Examples of these are patient-led partner referral (in which patients are asked to refer their sex partners for treatment) and patient-delivered partner treatment (in which participants are given antibiotics to deliver to sex partners), of which the acceptability and effectiveness have been documented in several studies [7–11]. Understanding factors related to partner referral is necessary to the success of partner-targeted interventions in preventing STIs; however, these factors have been insufficiently assessed in women who are diagnosed with a STI in Vietnam.

Although Vietnam has made remarkable progress in gender equity in the past decade, women are still inferior to men in some aspects such as the segregation of women into vulnerable jobs, less involvement in public life, lower health outcomes, being victims of domestic violence, and lack of ability in decision-making [12]. According to The Global Gender Gap Report in 2014, Vietnam ranks 76^th^ out of 142 countries in the overall gender gap index and ranks 137^th^ in the health and survival index [13]. Consequently, women have less power in communicating sexual and reproductive health issues with their partners [14], and thus may have low self-efficacy in partner notification or referral. For those women who come to a STI clinic and self-initiate treatment, very little is known about the proportions who feel sufficiently confident to inform their male partners about their STI positivity, convince partners to undergo STI screening, or give STI medications to partners. All of these factors would contribute to the effectiveness of partner-targeted interventions. Self-efficacy has been shown to be a strong predictor of behavioral performance [15–18] and increasing self-efficacy is a highly effective approach for STI prevention in women [18]. Therefore, assessing women’s self-efficacy in initiating STI-diagnosis-related communications with their partners may help to predict their future behavior with regard to such situations [17] and suggests the most likely causes of failure to follow through, which can inform behavior modification in future interventions [15]. Also of interest in designing partner-targeted interventions is the likelihood of male partners’ cooperation, e.g., to accompany females to an STI examination, to participate in STI treatment, and to take STI medications; however, information about the likelihood of these behaviors is scarce. Women’s and partners’ demographic and behavioral characteristics associated with women’s self-efficacy and perceived likelihood of their partners’ cooperation in STI treatment are also underexplored.

Given limited evidence and the high vulnerability of Vietnamese women to STIs due to their male partners’ risky behaviors [19], we studied women at high risk for STIs in Ho Chi Minh City (HCMC), Vietnam, to determine their self-efficacy in STI-diagnosis-related communications with their primary male partners and their perceptions of male partners’ likelihood to cooperate in STI treatment. We then assessed these results according to participant and partner demographic and behavioral characteristics. Focus was placed on women because of their lower power in gender relations, their reduced power in sexual communication, and consequently their higher vulnerability to reinfection. Moreover, the target settings for this study and for future interventions were the national obstetrics/gynecology hospitals in HCMC, where most of patients who come for STI screening and treatment are female.

## Methods

### Study design and setting

This study was nested within a clinic-based cross-sectional study that had the primary aim of examining the concordance of genital and oral HPV infection in women at high risk of STIs in HCMC. Participants were women who attended a national obstetrics/gynecology hospital in HCMC between August and October 2013.

### Procedures

The methods have been discussed in full elsewhere [3]. Briefly, a combined method of convenience and snowball sampling was employed. All eligible women were invited to participate. Women were eligible if they were 18–45 years of age and had *any one of the following* STI-related risks: (i) had ≥3 different sexual partners in their lifetime, (ii) had ≥2 different sexual partners in the past month, (iii) had been diagnosed with a bacterial or viral STI for ≥2 times or ≥2 bacterial or viral types of STI, including STI positivity identified at the time of recruitment, and including HIV or HPV infection, or (iv) had ever had sex in exchange for money or other goods (i.e., FSWs). Participants who were FSWs were asked to refer other potential FSWs in their network. Because sex work is illegal in Vietnam, a behavioral characteristic (e.g., having multiple sexual partners) is often used as a proxy measure to define FSW groups. In Vietnam, women are unlikely to have multiple sexual partners in their lifetime; for example a study reported that the mean lifetime number of sexual partners of women was 1.1 (*SD* = 0.5) [20]. Thus, our first two criteria of lifetime and recent numbers of sexual partners served as proxies to recruit women at higher risk for STIs.

Trained nurses and physicians obtained written informed consent and then conducted oral interviews in Vietnamese with participants in private clinic rooms. No potentially identifiable information was collected from participants. The study protocol was approved by the Committee for the Protection of Human Subjects, The University of Texas Health Science Center at Houston (HSC-SPH-13-0297), and the Ethical Review Committee, Tu Du Hospital (QD-BVTD-2424).

### Measures

The definition of “primary male partner,” shortened to “partner” in this report, was the male with whom participants most frequently had any type of sex during the previous 90 days. This partner could be a paying (e.g., “sweethearts”) or non-paying partner (e.g., your husband or boyfriends). We defined the primary partner based on frequencies of sexual activities because our future intervention aims to prevent STI recurrence in these women by notifying and/or giving medication to their regular partners, i.e., the partner with whom the women most frequently have sex. The primary variables were participants’ self-efficacy to disclose their STI positivity to primary male partners, to ask partners to have an STI examination or obtain treatment, and, if the STI was bacterial, to give STI medications to partners. Below, we refer to participants’ self-efficacy in these actions generally as “self-efficacy in STI-diagnosis-related communications”. Response options were based on a 5-point Likert scale. An example item is, “If you know you have an STI (including HIV), how confident are you that you can tell your primary partner about your STI-positive status?” Responses ranged from *1 = not confident at all* to *5 = very confident.* Partners’ likely cooperation, i.e., partners’ likelihood to get an STI examination and likelihood to take STI medications given by participants, was assessed by asking participants the questions “How likely do you think your primary partner will come to a clinic for an STI examination and treatment?” or “… take medications as instructed?”. Responses were also on a 5-point Likert scale, ranging from *1 = not likely at all* to *5 = very likely.*

Additional variables were partners’ frequency of having previously accompanied participants to gynecology clinics and participants’ ratio of actual past disclosure of STI positivity to their partners. Partners’ frequency of previously accompanying participants to gynecology clinics was measured for women who had ever had a gynecological visit for STI screening, using the question, “Of all the times you came to a gynecology clinic because of a possible STI, how many times did your primary partner come with you?” Responses ranged from *1 = never* to *5 = every time*. The ratio was calculated by dividing the number of times that participants had actually disclosed bacterial-STI positivity to their partners by the number of times that the participants were diagnosed with a bacterial STI.

Although our aim was mainly to describe participants’ self-efficacy and expected levels of partners’ cooperation, we additionally examined associations between these variables and both the participants’ and partners’ socio-demographic and behavioral characteristics. Focus was placed on each woman’s primary male sexual partner because women are found to be less likely to negotiate condom use and more likely to resume having sex with their primary partner while having STIs [21, 22]. Focusing on primary partners may facilitate information recall, as well. Participants’ characteristics assessed were age, education level (secondary school/lower vs. high school/higher) [23, 24], ever having traded sex [25], alcohol use, and drug use [25]. Partners’ characteristics assessed were partner type (husband/fiancé vs. boyfriend/other) [26, 27], partner age relative to the participant (about the same age/younger vs. ≥2 years older) [18], number of STI diagnoses (0 vs. ≥1), alcohol use, and drug use. Ever having traded sex among the women was defined as having any type of sex for money, drugs, or other in-kind exchange. Alcohol use was defined as having had at least one alcoholic drink (330 ml of beer [the typical can or bottle], 140 ml of wine [a full glass], or 40 ml of liquor) in the past 90 days [28]. Drug use was defined as any use of cocaine, heroin, or methamphetamine in the individual’s lifetime. These data were obtained through participants’ self-report.

### Analysis

Frequencies and percentages were used to provide descriptive statistics for each main variable. We used the Kruskal-Wallis test to examine associations between main ordinal variables and binary or ordinal participants’ and partners’ demographic/behavioral characteristics. For multivariable analyses, we chose to dichotomize the dependent variables into “slightly confident” or “very confident” (i.e., a score of 4 or 5 on the Likert scale), which denoted the participants’ confidence, versus the others (i.e., a score of 1–3 on the Likert scale). We deemed that this dichotomization was the most appropriate way for the multivariable analyses of our data because (i) the skewed distribution of self-efficacy scales (Table 1) violated the assumptions of parametric methods such as analysis of variance, and (ii) the qualitative and unequal intervals within a Likert scale hindered the applicability of ordinal logistic regression method. We used multivariable binary logistic regression models to examine the adjusted associations between main self-efficacy outcomes and some participants’ characteristics. Variable selection for multivariable models was based on *a priori* knowledge (e.g., known correlated factors in literature review), following criteria for control of confounding [29], and actual associations in bivariate analyses in our data. Analyses were performed with STATA version 11.0 (StataCorp LP, College Station, Texas, USA). Participants with missing values were excluded from analyses. A two-sided *P* value of .05 or less was considered significant.

**Table 1:** Participants’ self-efficacy in STI-diagnosis-related communications and perceived likelihood of primary male partners’ cooperation

Main variable	No.	%
Participants’ self-efficacy in disclosing their STI positivity to primary male partner (*n* = 118)		
Not confident at all	20	17.0
Slightly not confident	4	3.4
So-so	11	9.3
Slightly confident	15	12.7
Very confident	68	57.6
Participants’ self-efficacy in asking primary male partner to have an STI examination (*n* = 116)		
Not confident at all	19	16.4
Slightly not confident	7	6.0
So-so	18	15.5
Slightly confident	19	16.4
Very confident	53	45.7
Participants’ self-efficacy in giving medications for STI treatment to primary male partner (*n* = 116)		
Not confident at all	5	4.3
Slightly not confident	5	4.3
So-so	26	22.4
Slightly confident	35	30.2
Very confident	45	38.8
Primary male partners’ likelihood to get an STI examination (*n* = 105)		
Not likely at all	20	19.1
Unlikely	15	14.3
Maybe	26	24.8
Likely	27	25.7
Very likely	17	16.2
Primary male partners’ likelihood to take medications for STI treatment given by the participants (*n* = 116)		
Not likely at all	5	4.3
Unlikely	5	4.3
Maybe	26	22.4
Likely	35	30.2
Very likely	45	38.8
Primary male partners’ frequency of accompanying participants to gynecological clinics (*n* = 89)^a^		
Never	48	53.9
< 1/2 the time	18	20.2
= 1/2 the time	7	7.9
> 1/2 the time	7	7.9
Every time	9	10.1

^a^Among those who had previously visited a gynecology clinic due to an STI symptom

## Results

Of the 129 eligible women, 126 (98.0 %) agreed to participate. The most common reason for refusal was time conflict. Participants’ characteristics were described previously [3]. Briefly, the mean age of the 126 participants was 31.9 years (*SD* = 6.2). About half had a high school education or higher (i.e., ≥10^th^ grade), and the majority (74.6 %) lived in city or urban areas. Over a third (37.3 %) had ever traded sex. All participants had been diagnosed with an STI (including both bacterial and viral STIs) at least one time previously; 20.6 % (26/126) had ever been diagnosed with a bacterial STI. Among all participants, 70.6 % (89/126) had ever visited a gynecology clinic due to an STI symptom. Approximately two-thirds of the women’s primary sexual partners were husbands or fiancés (22.9 % in self-identified FSWs and 77.1 % in the other participants). Nearly three-fourths of the primary partners were at least two years older than the participants and about two-thirds of the partners were at least five years older than the participants.

The distribution of outcomes of interest is displayed in Table 1. The proportions of participants who were slightly or very confident that they could disclose their STI positivity to primary partners, to ask partners to have an STI examination or obtain treatment, and to give STI medications to partners were 70.3 %, 62.1 %, and 69.0 %, respectively. According to participants’ estimates, the proportions of partners who would be likely or very likely to have an STI examination and to take STI medications were 41.9 % and 69.0 %, respectively. Among women who were ever diagnosed with any bacterial STI, the mean ratios of past STI disclosure were 0.63 (*SD* = 0.5) overall (*n* = 26), 0.70 (*SD* = 0.4) among those who ever traded sex (*n* = 12), and 0.43 (*SD* = 0.4) among those who never traded sex (*n* = 14). A total of 34.6 % (9/26) had not disclosed their positive results to their partners (data not shown). About 54.0 % of participants reported that their partners had never accompanied them to gynecology clinics for STI treatment.

We next assessed the characteristics of participants and partners that were associated with women’s self-efficacy and perceived likelihood of partner cooperation. Participants who had a low education level (≤ secondary school) or had ever traded sex had lower self-efficacy levels in general (Table 2). Women with partners who were husbands or fiancés or who used drugs generally had increased self-efficacy scores. Women’s self-efficacy did not differ by partners’ number of STI diagnoses (i.e., 1 or >1) or by partner’s age difference (≥2 years). Analyses between participants who had a partner of the same age or younger or <5 years older and participants who had a partner ≥5 years older were also non-significant (*P* values for the three self-efficacy variables were ≥ .20, data not shown). Further analyses among women who had only one lifetime male partner generated the same result (*P* values for the three self-efficacy variables were ≥ .29, data not shown). Ratios of past disclosure of STI positivity did not differ by self-efficacy of disclosing their STI positivity to primary male partner (*P* value = .053). Results of the multivariable models suggested that women’s ever traded sex status and lower education level were still significantly associated with lower self-efficacy in STI-related communications and medication delivery; while partner type might be not (Table 3). Partners’ cooperation (i.e., likelihood to get an STI examination or to take STI medications given by participants) was perceived to be significantly more likely when partners were husbands or fiancés (Table 4).

**Table 2:** Mean scores for participants’ self-efficacy in STI-diagnosis-related communications by participants’ characteristics and Kruskal-Wallis test results

Characteristic	Total (n)	Participants’ self-efficacy		
		Mean score on a scale from 1 to 5		
		Disclosing their STI positivity to primary male partner	Asking primary male partner to have an STI examination	Giving medications for STI treatment to primary male partner
Participants’ education level				
Secondary school or lower	61	3.61	3.45	3.78
High school or higher	64	4.23	3.95	4.41
*P* value		.03	.04	.005
Participants’ ever having traded sex				
Yes	47	2.90	3.20	3.40
No	79	4.42	3.96	4.53
*P* value		.005	<.001	.002
Participants’ alcohol use				
Yes	62	4.14	3.95	4.27
No	64	3.68	3.39	3.93
*P* value		.12	.10	.11
Participants’ drug use				
Yes	16	3.79	4.21	3.75
No	107	3.95	3.66	4.17
*P* value		.60	.46	.09
Partner type				
Husband/fiancé	86	4.31	4.02	4.45
Boyfriend/other	37	2.97	2.87	3.31
*P* value		<.001	<.001	<.001
Partners’ age				
About the same age/younger	35	4.38	3.94	4.03
≥ 2 years older	90	3.70	3.58	4.12
*P* value		.05	.35	.67
Partners’ number of STI diagnoses				
0	34	4.53	4.29	4.68
≥ 1	9	4.22	3.75	4.44
*P* value		.20	.27	.87
Partners’ alcohol use				
Yes	94	4.22	3.73	3.96
No	23	3.84	3.71	4.16
*P* value		.34	.89	.40
Partners’ drug use				
Yes	11	4.11	3.79	4.22
No	102	2.82	3.17	3.67
*P* value		.03	.13	.03
Ratio of past disclosing participants’ STI positivity to primary male partner^a^				
*P* value		.05	-	-

^a^Number of times disclosed to primary male partner divided by number of times diagnosed with a bacterial STI, among women ever diagnosed with any bacterial STI (*n* = 26); range, 0–1

**Table 3:** Adjusted associations between participants’ self-efficacy in STI-diagnosis-related communications and selected participants’ characteristics in multivariable logistic regression models

Characteristics	Participants’ self-efficacy^a^		
	Odds ratios (95 % confidence intervals)		
	Disclosing their STI positivity to primary male partner	Asking primary male partner to have an STI examination	Giving medications for STI treatment to primary male partner
Participants’ education level			
Secondary school or lower	1	1	1
High school or higher	2.14 (0.89–5.45)	1.73 (0.75–4.01)	2.55 (1.02–6.39)
*P* value	.08	.20	.05
Participants’ ever having traded sex			
No	1	1	1
Yes	0.25 (0.09–0.69)	0.54 (0.21–1.41)	0.31 (0.11–0.89)
*P* value	.008	.21	.02
Partner type			
Husband/fiancé	1	1	1
Boyfriend/other	0.47 (0.16–1.31)	0.62 (0.24–1.58)	0.43 (0.16–1.15)
*P* value	.15	.32	.09

^a^All dependent variables were dichotomized as “slightly confident” or “very confident” (i.e., a score of 4 or 5 on the Likert scale) versus the others (i.e., a score of 1–3 on the Likert scale)

**Table 4:** Mean scores for likelihood of primary male partners’ cooperation by partners’ characteristics and Kruskal-Wallis test results

Partners’ characteristic	Mean score on a scale from 1 to 5	
	Primary male partners’ likelihood to get an STI examination	Primary male partners’ likelihood to take medications for STI treatment given by the participants
Partner type		
Husband/fiancé	3.25	4.07
Boyfriend/other	2.61	3.61
*P* value	.03	.04
Partners’ age		
About the same age/younger	3.14	3.75
≥ 2 years older	3.03	4.02
*P* value	.72	.31
Partners’ number of STI diagnoses		
0	3.16	4.21
≥ 1	2.89	3.78
*P* value	.62	.31
Partners’ alcohol use		
Yes	3.08	3.96
No	3.08	3.97
*P* value	.81	.76
Partners’ drug use		
Yes	3.03	4.02
No	3.30	3.58
*P* value	.60	.12

## Discussion

Our results showed that women’s self-efficacy in STI-diagnosis-related communications was fairly high, with at least 60.0 % of women “slightly confident” or “very confident” about each of the three interactions. Their perceptions of their primary male partners’ likelihood to follow up on STI examination or treatment were low for the partners’ taking an STI examination (41.9 %) but fairly high for partners’ taking an STI medication given by the participant (69.0 %).These results suggest a potential platform for piloting or implementing patient-led partner-targeted STI interventions in HCMC.

The proportions of participants who were “very confident” that they could disclose their STI positivity to primary partners, to ask the partners to have an STI examination or obtain treatment, and to give STI medications to partners were 57.6 %, 45.7 %, and 38.8 %, respectively. In comparison, Hoffman et al. (2008) reported that 74.6 % of African American immigrants and 85.6 % of African American US-born women were extremely confident that they could notify their regular partners that they had an STI, and 63.5 % of African American immigrants and 76.7 % of African American US-born women were extremely confident that they could convince their regular partners to obtain STI screening [27]. A reason that the proportions were low in our study may be that in Vietnam, women’s status is still seen as inferior to men’s, especially FSWs, which leads to women’s lower power and hence low self-efficacy to discuss their sexual health [14, 30, 31] or to ask their male partners to get an STI examination. Thus, improving women’s self-efficacy should be a component in partner-targeted management approaches for STI control in Vietnam.

Although the majority of participants showed fairly high self-efficacy in STI-diagnosis-related communications, only 16.2 % and 38.8 % perceived that their primary partners would be “very likely” to have an STI examination or to take given STI medications, respectively. Therefore, to effectively implement and broaden couple-based STI interventions in Vietnam (e.g., self-referral or patient-delivered partner therapy), health education programs should aim to raise both participants’ and partners’ awareness of the necessity of treating all regular sexual partners. In addition, future studies are needed to further explore why surveyed women perceived a low likelihood of partners’ cooperation with STI examination or treatment, and future partner-targeted STI interventions should address these factors to be effective.

Women’s self-efficacy in STI-diagnosis-related communications with their male partners was associated with some individual characteristics. Women who had a high school education or higher potentially had higher self-efficacy for the examined behaviors. Similar results have been found for African American adolescent women’s self-efficacy to discuss STI testing with their male partners [23, 24]. This may be because higher education diminishes gender inequality and develops women’s power and confidence in communicating about such sensitive issues [32]. Ever having traded sex was inversely associated with the women’s self-efficacy. Previous studies reported low self-efficacy in condom use and birth control among American FSWs [25]. FSWs’ low self-efficacy might be attributable to several reasons. First, they might perceive that owing to their multiple concurrent relationships, they would be more likely to be infected with an STI first, and then transmit it to their regular partners. Fear of being accused of being a main source of STI acquisition and transmission was reported as undermining women’s confidence to disclose their STI positivity to partners [33]. Second, due to stigma and marginalization, FSWs might receive less social support, and hence their sex-related communication with other people, not only with their main partners, might be hampered [34]. Finally, primary partners’ unawareness of women’s sex-worker status would be a major barrier for STI-positivity disclosure.

With regard to partners’ characteristics, the women’s self-efficacy in STI-diagnosis-related communications was generally higher when the partner was a husband or fiancé, indicating a more intimate relationship. Some previous studies have found a similar association [26, 27]. These associations, however, did not remain statistically significant in the adjusted analysis, which might be due to a small number of participants in subgroups. If these non-associations in the multivariable model were true and were not due to a small sample size, this result suggests that partner-targeted interventions can be feasible for both types of partners. Several factors may explain the association between partners’ drug use and women’s increased self-efficacy. For example, women might perceive that their risk of infection came from the drug-using partners, and hence women were more confident to communicate with their partners. Alternatively, this association might be confounded by another factor, for which we could not control in our study, such as a shared risk or a shared HIV-positive status in these couples. Partners’ age was not significantly associated with women’s self-efficacy, ratio of past STI disclosure, or perceived likelihood of partners’ cooperation. This finding is unlike those of previous studies that have shown that having an older partner may lead to lower women’s self-efficacy in making safe-sex decisions or practicing safe-sex, potentially because of a power imbalance caused by the age differential [18]. Also noteworthy, women’s self-efficacy in STI-diagnosis-related communications did not differ by partners’ number of STI diagnoses, even among women who had only one lifetime male partner. These results imply that even when women realized that their primary partner was a source of STI acquisition and transmission (since women with one lifetime male partner could not (re)acquire an STI from someone else), their confidence about revealing their STI positivity did not change. Further elucidation of women’s barriers to disclosure in this circumstance is needed.

A moderate proportion of past STI notification (65.4 % of 26 women who were ever diagnosed with a bacterial STI), comparable to those of other studies [26, 35, 36], suggests the applicability and feasibility of partner notification approaches in Vietnam. For women who have multiple concurrent sexual partners or who are FSWs, notification and treatment of as many partners as possible--the primary partner at minimum--is important.

Some study limitations should be noted. Our findings may not be generalizable to other populations due to the convenience and snowball sampling methods; however, these sampling techniques made it practical and feasible to reach a population at high risk for STIs [37], which should receive priority for interventions. Our data might be subject to recall bias, under-reporting, and social desirability bias. The reliability of the self-report may be compromised for responses related to risk behaviors of participants’ male sexual partners. However, a study of partners’ risk behaviors at a San Francisco STI clinic found STI patients’ and their regular partners’ responses exhibited moderate to substantial consensus [38]. Therefore, the self-report of one partner could accurately reflect risk behaviors of the other partner in the relationship. To collect accurate data in a population with a low-to-middle education level, we decided to use an in-person interview approach so that the questions could be further explained to participants, if needed. This might have led to some bias in self-reporting sensitive behaviors. As described above, several structural gender disparities such as economic dependence or risk of domestic violence might have further constrained women’s self-efficacy in partner notification or partner referral. These factors were beyond the scope of this study; further study is needed to examine these. Moreover, possibly due to the limited sample size of participants who ever had a bacterial STI diagnosis (*n* = 26), we did not find any significantly associated characteristics for those who previously disclosed their STI positivity to partners. Lastly, our study did not have a sufficient sample size to control for other possible confounders or to perform stratified analyses (e.g., by traded-sex status or by paying- versus non-paying partners)*.* Further studies are needed to explore this issue more deeply in separate groups of women.

Despite these limitations, to our knowledge, this is the first study to assess women’s self-efficacy in STI disclosure, partner referral, and treatment delivery in Vietnam. Another strength of this study is that we examined both participants’ self-efficacy and their perception of partners’ cooperation, which are interrelated. Women who have high self-efficacy but low expectation of partners’ cooperation may be less likely to disclose their STI positivity to partners, to ask partners to have an STI examination, or to give STI treatment medications to partners, compared to those who have high self-efficacy and high expectation of their partners’ cooperation. Thus, our study provides useful information for developing future partner-targeted STI management programs in Vietnam.

## Conclusions

Results of this study showed that women’s self-efficacy in disclosing their STI positivity to primary male partners, in asking the partners to have an STI examination or treatment, and in delivering STI treatment medications to the partners were fairly acceptable. These results suggest a potential platform for piloting or implementing partner-targeted STI interventions in HCMC, Vietnam. Nevertheless, these women’s self-efficacy levels were not yet optimal for a success of these types of programs. In addition, surveyed women perceived a low likelihood of partners’ cooperation in STI examination or treatment. Thus, future studies are needed to further explore why it was so and future partner-targeted STI interventions should address these factors in order to be effective and successful.

## Abbreviations

STI: Sexually transmitted infection
FSW: Female sex worker
HCMC: Ho Chi Minh City
